# A 265-Nanometer High-Power Deep-UV Light-Emitting Diode Rapidly Inactivates SARS-CoV-2 Aerosols

**DOI:** 10.1128/msphere.00941-21

**Published:** 2022-03-17

**Authors:** Hiroshi Ueki, Mutsumi Ito, Yuri Furusawa, Seiya Yamayoshi, Shin-ichiro Inoue, Yoshihiro Kawaoka

**Affiliations:** a Department of Virology, Institute of Medical Science, University of Tokyo, Tokyo, Japan; b Center for Global Viral Diseases, National Center for Global Health and Medicine, Tokyo, Japan; c Laboratory of Ultrastructural Virology, Institute for Frontier Life and Medical Sciences, Kyoto University, Kyoto, Japan; d Advanced ICT Research Institute, National Institute of Information and Communications Technology (NICT), Kobe, Japan; e Department of Special Pathogens, International Research Center for Infectious Diseases, Institute of Medical Science, University of Tokyo, Tokyo, Japan; f Department of Pathobiological Sciences, School of Veterinary Medicine, University of Wisconsin-Madison, Madison, Wisconsin, USA; Mount Sinai School of Medicine

**Keywords:** COVID-19, LED, SARS-CoV-2, aerosols, deep UV

## Abstract

Severe acute respiratory syndrome coronavirus 2 (SARS-CoV-2) infection (COVID-19) is an acute respiratory infection transmitted by droplets, aerosols, and contact. Controlling the spread of COVID-19 and developing effective decontamination options are urgent issues for the international community. Here, we report the quantitative inactivation of SARS-CoV-2 in liquid and aerosolized samples by a state-of-the-art, high-power, AlGaN-based, single-chip compact deep-UV (DUV) light-emitting diode (LED) that produces a record continuous-wave output power of 500 mW at its peak emission wavelength of 265 nm. Using this DUV-LED, we observed a greater-than-5-log reduction in infectious SARS-CoV-2 in liquid samples within very short irradiation times (<0.4 s). When we quantified the efficacy of the 265-nm DUV-LED in inactivating SARS-CoV-2, we found that DUV-LED inactivation of aerosolized SARS-CoV-2 was approximately nine times greater than that of SARS-CoV-2 suspension. Our data demonstrate that this newly developed, compact, high-power 265-nm DUV-LED irradiation system with remarkably high inactivation efficiency for aerosolized SARS-CoV-2 could be an effective and practical tool for controlling SARS-CoV-2 spread.

**IMPORTANCE** We developed a 265-nm high-power DUV-LED irradiation system and quantitatively demonstrated that the DUV-LED can inactivate SARS-CoV-2 in suspensions and aerosols within very short irradiation times. We also found that the inactivation effect was about nine times greater against aerosolized SARS-CoV-2 than against SARS-CoV-2 suspensions. The DUV-LED has several advantages over conventional LEDs and mercury lamps, including high power, compactness, and environmental friendliness; its rapid inactivation of aerosolized SARS-CoV-2 opens up new possibilities for the practical application of DUV-LEDs in high-efficiency air purification systems to control airborne transmission of SARS-CoV-2.

## INTRODUCTION

Severe acute respiratory syndrome coronavirus 2 (SARS-CoV-2) infection (COVID-19), which was first detected in China at the end of 2019, rapidly spread worldwide, prompting the World Health Organization (WHO) to declare a pandemic on 11 March 2020. As of October 2021, the number of infected people worldwide has exceeded 238 million, of whom approximately 4.9 million have died (COVID-19 Map; Johns Hopkins Coronavirus Resource Center, https://coronavirus.jhu.edu/map.html). The main infectious routes of COVID-19 are considered to be droplet, aerosol, and contact transmission (1). Ethanol and sodium hypochlorite have been used to disinfect surfaces of objects contaminated by microbes including SARS-CoV-2, because they have shown remarkable inactivation effects. However, disinfection methods for objects that are sensitive to liquids and effective methods for inactivating virus in aerosols have not been established. Therefore, there are still infection control issues that need to be addressed.

Deep-UV (DUV) light-emitting diodes (LEDs) have attracted attention because their properties could be useful in technologies that provide a chemical-free approach to virus inactivation. The DUV region is usually defined as the wavelength range from 200 nm to 300 nm. Photochemical inactivation of viruses and microorganisms is most effective with UV-C photons in the 100- to 280-nm wavelength range with a peak effectiveness near 265 nm, which is the approximate absorption maximum wavelength for DNA and RNA (2, 3). UV light in the longer wavelength range, classified as UV-A (320 to 400 nm) and UV-B (280 to 320 nm), also inactivates SARS-CoV-2; however, the efficiency is relatively low compared with that of UV-C (4, 5). It has been reported that far-UV-C (222 nm) produced by excimer lamps has potential for SARS-CoV-2 inactivation, but a long irradiation time would be required for viral inactivation (99.7% inactivation in 30 s) due to the light output of excimer lamps being relatively low (6). A recent study showed that UV-C illumination prevents SARS-CoV-2 airborne transmission in a hamster model (7); however, no quantitative analysis of the relationship between the irradiation dose of the UV-C lamp or DUV-LED and the photosensitivity of aerosolized SARS-CoV-2 has been performed to our knowledge. DUV-LEDs that operate at the peak emission wavelength of 265 nm offer significant advantages over conventional gas-discharge lamps that radiate UV-C light (e.g., mercury vapor lamps, which are commonly used to perform irradiation at a wavelength of 254 nm); these advantages include compactness and flexibility (which are useful for device design), emission wavelength diversity (i.e., the ability to be set at the effective wavelength [265 nm] for disinfection), single-peak emission, low drive voltage requirements, zero required warmup time, and environmental friendliness (8–10). Furthermore, because the manufacture, import, and export of products containing mercury have been prohibited since 2020 under the Minamata Convention on Mercury (http://www.mercuryconvention.org/), which is an international treaty intended to protect both human health and the environment, alternatives to mercury lamps are being sought.

Despite their high-technology potential, and the strenuous efforts that have gone into their development (11, 12), the aluminum gallium nitride (AlGaN)-based DUV-LEDs reported to date have much lower optical output powers than mercury lamps. The typical output power from commercially available high-power 265-nm DUV-LEDs is approximately 50 mW. Recently, researchers reported SARS-CoV-2 inactivation effects by DUV-LEDs with low irradiation power densities (i.e., 3.75 mW/cm^2^) (13); in their study, virus infectivity was reduced by only 1 log unit with 1-s irradiation in a liquid, which is not efficient enough for practical use. Additionally, in their study, the experimental conditions under which the inactivation performances were evaluated were limited to the use of petri dishes with small liquid samples containing viruses diluted in culture medium (13). As yet, neither the inactivation of aerosolized SARS-CoV-2 by a DUV-LED nor the quantitative effectiveness of such a device for this application has been reported. Because SARS-CoV-2 can remain infectious in aerosols and can thus be transmitted through them (14), it is essential to find efficient ways of inactivating infectious viruses in aerosols. Notably, in evaluations of liquid samples, the effectiveness of inactivation is strongly affected by the applied liquid culture medium because culture media typically absorb DUV light (15). An accurate determination of the effectiveness of aerosolized SARS-CoV-2 inactivation by a 265-nm DUV-LED will provide essential information for the development of practical infection-prevention technologies that can be used against aerosolized SARS-CoV-2; the goal of this study was to do that.

## RESULTS

**FIG 1:**
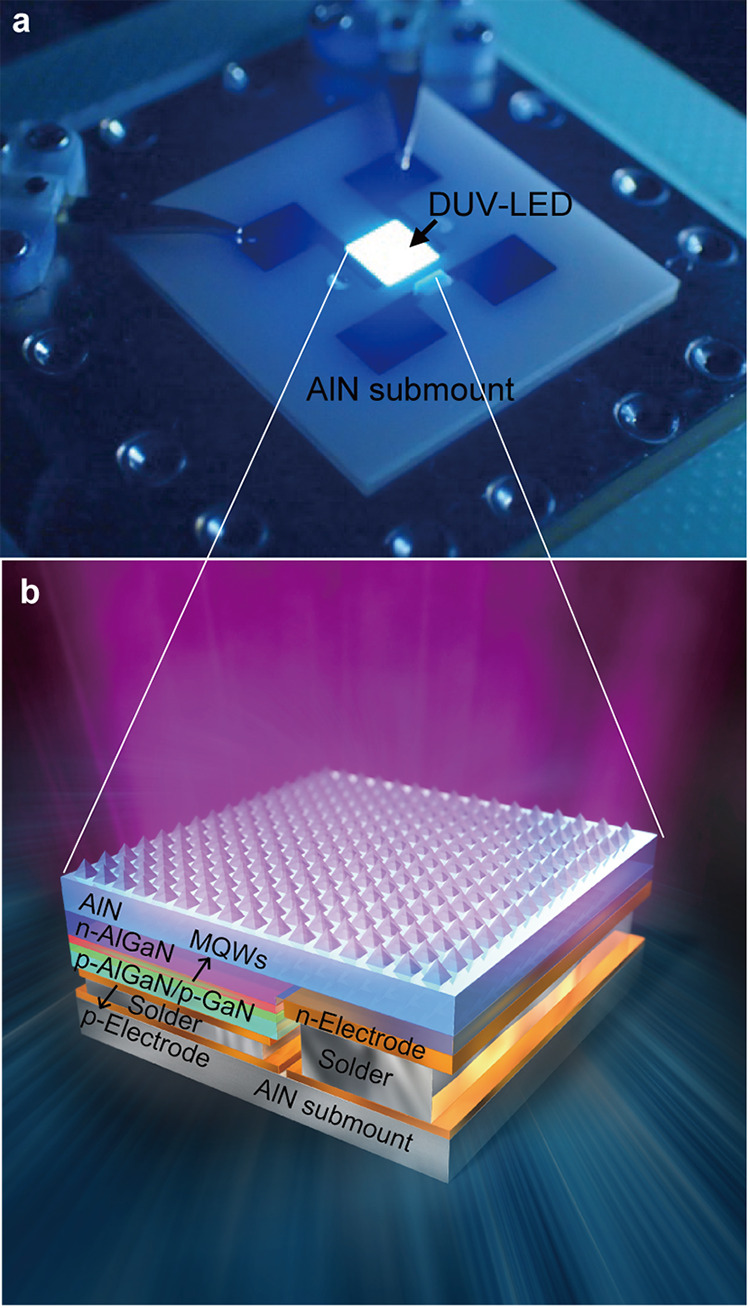
DUV-LED irradiation system used in this study. (a) Photograph of the single-chip DUV-LED mounted on an AlN submount and a heat sink. (b) Schematic of the DUV-LED layer structure based on AlGaN multiple quantum wells (MQWs) with its large-area AlN nanophotonic light extraction structure.

Figure 1a shows the DUV-LED irradiation system that we developed and used in the present study; this system comprises a single-chip DUV-LED flip-chip mounted on an aluminum nitride (AlN) submount and a heat sink. The main causes of the low output power of current AlGaN-based DUV-LEDs are the high defect densities (i.e., high dislocation densities) that occur in their active layers and the extremely low light extraction efficiency of the as-grown devices (2, 11, 12). To overcome these problems, we previously demonstrated a pseudomorphic AlGaN-based DUV-LED fabricated on an AlN substrate with a nanophotonic light extraction structure (16, 17). Early power saturation (known as efficiency droop) at high-injection current densities is also a common problem for flip-chip mesa-type DUV-LEDs. Therefore, to achieve high-current operation and increase the output flux per single-chip LED by reducing the device current density, we fabricated a 265-nm flip-chip AlGaN-based DUV-LED with large-area AlN nanophotonic light extraction structures and larger emitting areas (chip size, 1.8 by 1.8 mm^2^) (cf. the emitting areas of previous devices [chip size, 1 by 1 mm^2^]), as illustrated schematically in Fig. 1b. The techniques used for the LED fabrication processes are described in greater detail elsewhere (17).

**FIG 2:**
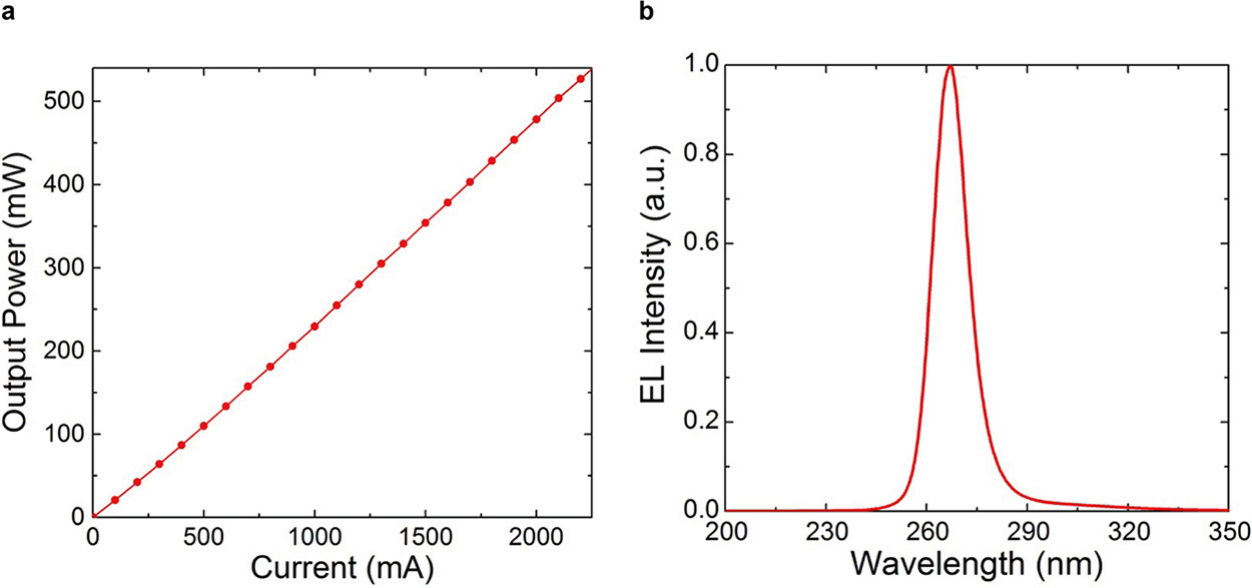
DUV-LED device performance. (a) Output power characteristic as a function of the continuous injection current for the DUV-LED with the large-area AlN nanophotonic light extraction structure. (b) Electroluminescence (EL) spectrum of the LED with the AlN nanophotonic light extraction structure. a.u., arbitrary unit.

The output power characteristics of the fabricated DUV-LED as a function of the injection current are shown in Fig. 2a. Output powers of more than 500 mW were observed from the DUV-LED under continuous-wave conditions at room temperature. This is the highest output power reported to date for a single-chip DUV-LED in the UV-C (wavelength < 280 nm) regime. A nearly symmetric, single emission peak was also observed at approximately 265 nm with a full-width at half-maximum (FWHM) of 12 nm for the DUV-LED (Fig. 2b). An irradiation power density of 54 mW/cm^2^ was recorded at the sample position for use in SARS-CoV-2 inactivation.

**FIG 3:**
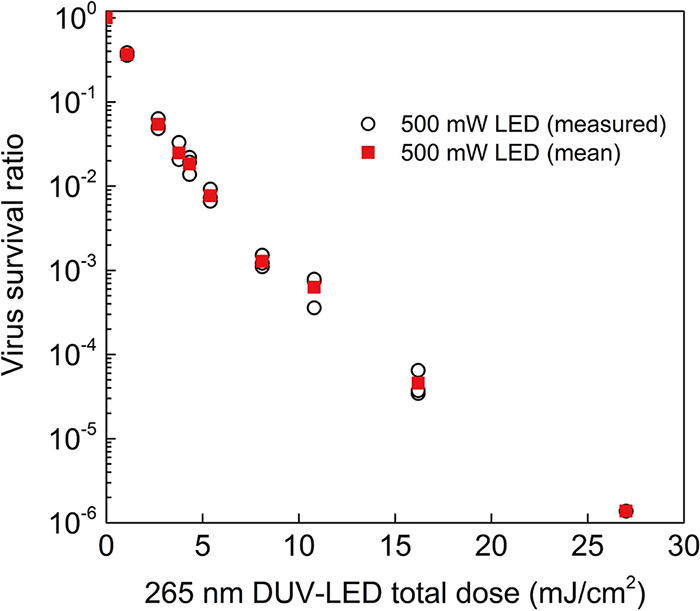
DUV-LED inactivation of SARS-CoV-2 suspension. White circles indicate values measured for each experiment (*n* = 3) when using the high-power 500-mW DUV-LED, and red squares indicate the mean values. Significant differences were observed in each irradiated group compared to the nonirradiated group (*P* < 0.05).

To investigate the inactivation effect of the DUV-LED on SARS-CoV-2, 80 μL of SARS-CoV-2 suspension (with 1% bovine serum albumin [BSA]) was spread evenly in a circular shape and immediately irradiated with the DUV-LED light from directly above at an irradiance of 54 mW/cm^2^ and a working distance of 55 mm (see Fig. S1 in the supplemental material). The virus suspension was immediately collected after LED irradiation and assessed by use of a plaque assay to determine the viral titer. The infectivity of the viruses was reduced to 1/1,000 after 0.167 s, 1/10,000 after 0.27 s, and 1/100,000 after 0.387 s of DUV-LED irradiation of the virus suspension; the exposure doses at each time point were 9.02 (mJ/cm^2^), 14.58 (mJ/cm^2^), and 20.90 (mJ/cm^2^), respectively (Fig. 3 and Table S1). *D*_99.9_ (i.e., the total dose required to inactivate 99.9% of the virus) for the SARS-CoV-2 suspension by the DUV-LED light was 9.02 mJ/cm^2^. The virus survival ratio after irradiation with the 500-mW DUV-LED is 10 times lower than an estimated corresponding ratio after irradiation with a conventional 50-mW DUV-LED.

10.1128/msphere.00941-21.1 FIG S1 (a) Photograph of the DUV-LED irradiation device. The DUV-LED unit was positioned on a stage whose height could be adjusted in the vertical direction. The LED light was focused by two collimator lenses, and the height of the sample stage was adjusted to irradiate samples. By driving the DUV-LED unit at a light output of 500 mW, the sample irradiation surface could be irradiated with an irradiance of 54 mW/cm^2^ (diameter, 20 mm). (b) Photograph of the SARS-CoV-2 suspension spread on a glass slide. Download FIG S1, TIF file, 14.4 MB. Copyright © 2022 Ueki et al. 2022 Ueki et al. https://creativecommons.org/licenses/by/4.0/ This content is distributed under the terms of the Creative Commons Attribution 4.0 International license.

**FIG 4:**
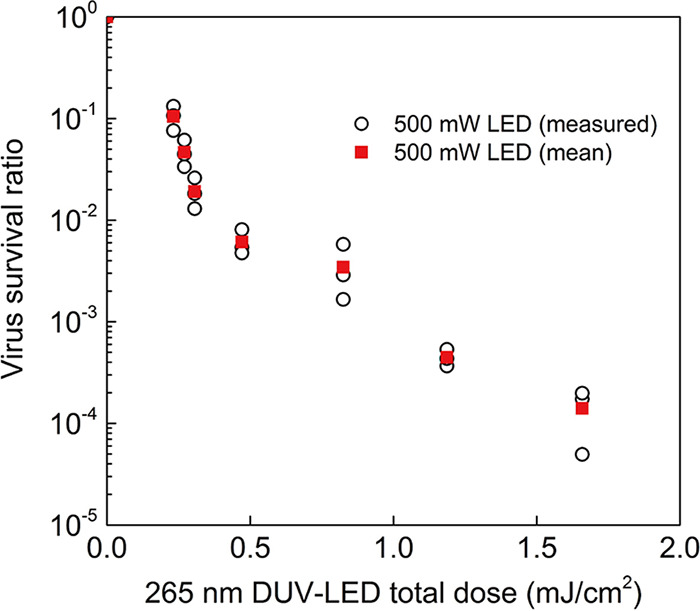
DUV-LED inactivation of SARS-CoV-2 aerosol. White circles indicate values measured for each experiment (*n* = 3) when using the high-power 500-mW DUV-LED, and red squares indicate the mean values. Significant differences were observed in each irradiated group compared to the nonirradiated group (*P* < 0.05).

10.1128/msphere.00941-21.4 TABLE S1 The irradiation time (s) or total dose (mJ/cm^2^) of the DUV-LED required to obtain the indicated viral survival rate (%) estimated by interpolations of the experimental mean values in Fig. 3 and 4. SARS-CoV-2 suspension (2.5 × 10^7^ PFU/mL with 1% BSA) was spread uniformly in a circular pattern on a glass slide, collected immediately after LED irradiation, and evaluated for the viral titer. The irradiation time (s) or total dose (mJ/cm^2^) of the DUV-LED required to obtain the indicated viral survival rate (%). SARS-CoV-2 aerosols were sprayed by using a nebulizer charged with the virus suspension (2.5 × 10^7^ PFU/mL with 1% BSA) into the chamber and collected with the air sampler while being irradiated with the DUV-LED light. The membrane in which the virus particles were trapped was immediately dissolved in DMEM, and then the virus infectivity was assessed by using a plaque assay. Download Table S1, PDF file, 0.2 MB. Copyright © 2022 Ueki et al. 2022 Ueki et al. https://creativecommons.org/licenses/by/4.0/ This content is distributed under the terms of the Creative Commons Attribution 4.0 International license.

To investigate the effect of DUV-LEDs on SARS-CoV-2 aerosols, we developed a test chamber for generating viral aerosols in a biosafety cabinet at our biosafety level 3 facility (Fig. S2). The SARS-CoV-2 aerosols generated in the chamber were collected with an air sampler by passing the virus through a DUV-transparent synthetic quartz tube. By using an optical particle counter to measure the size of wafting particles in the chamber, we determined that >94.9% of the particles were <2 μm in optical diameter and were, therefore, considered to be aerosols (Table S2).

10.1128/msphere.00941-21.2 FIG S2 Schematic image (a) and photograph (b) of the DUV-LED irradiation system for the SARS-CoV-2 aerosol. The test chamber was made of acrylic panels and constructed in a biosafety cabinet at a biosafety level 3 facility. One side plate of the chamber was connected to a customized compressor nebulizer and released a mist of virus suspension. The other side was connected to an air sampler through a synthetic quartz tube (center diameter, 20 mm) with high light transmission. The aerosols wafting through the chamber were passed through the synthetic quartz tube and trapped by the gelatin membrane of the air sampler. Aerosols passing through the synthetic quartz tube were irradiated with DUV-LED light from outside the tube. Download FIG S2, TIF file, 16.2 MB. Copyright © 2022 Ueki et al. 2022 Ueki et al. https://creativecommons.org/licenses/by/4.0/ This content is distributed under the terms of the Creative Commons Attribution 4.0 International license.

10.1128/msphere.00941-21.5 TABLE S2 A representative data set for particle size distribution of the wafting particles in the test chamber. The wafting particles in the test chamber were measured with a particle counter 5 min after the nebulizer began spraying. Download Table S2, PDF file, 0.2 MB. Copyright © 2022 Ueki et al. 2022 Ueki et al. https://creativecommons.org/licenses/by/4.0/ This content is distributed under the terms of the Creative Commons Attribution 4.0 International license.

To assess the effect of the LED light on the SARS-CoV-2 aerosols, the virus aerosols passing through the synthetic quartz tube were irradiated with DUV-LED light from outside the tube. Since the time for the virus aerosol to pass through the irradiation area of the LED light changes depending on the suction flow rate of the air sampler, the DUV irradiation doses supplied to the aerosolized SARS-CoV-2 were controlled by varying the flow rate. The velocity of the aerosols passing through the tube was measured by particle image velocimetry. When the suction volume of the air sampler was 90 L/min, 75 L/min, 57 L/min, 38 L/min, 25 L/min, 15 L/min, and 10 L/min, the average velocity of the aerosols in the tube was 4.65 m/s, 3.99 m/s, 3.23 m/s, 2.29 m/s, 1.34 m/s, 0.91 m/s, and 0.65 m/s, respectively (Movie S1 [see the video first to assist in understanding] and Fig. S3). Aerosolized SARS-CoV-2 was rapidly inactivated by DUV-LED irradiation to 1/10 after 0.0043 s (0.23 mJ/cm^2^), 1/100 after 0.0074 s (0.40 mJ/cm^2^), and 1/1,000 after 0.019 s (1.04 mJ/cm^2^) (Fig. 4 and Table S1). The biphasic pattern in Fig. 4 indicates that the rate of inactivation decreases much more slowly after an initial, more rapid exponential decay. This phenomenon is called the “tailing effect.” In fact, it can be seen in both Fig. 3 and 4. The tailing effect may be caused by light shielding due to viral particle aggregates or the presence of resistant viral subpopulations (18). The total dose required for *D*_99.9_ was 1.04 mJ/cm^2^ for SARS-CoV-2 aerosols, indicating that DUV-LED irradiation was approximately nine times more effective against virus aerosols than against the virus suspension. Previous reports on the inactivating effect of far-UV-C (222-nm) irradiation on aerosolized human coronaviruses or SARS-CoV-2 in solvent suggest that aerosolized human coronaviruses (1.2 to 1.7 mJ/cm^2^ for 99.9% inactivation) are more sensitive to UV light than SARS-CoV-2 in solvent (3 mJ/cm^2^ for 99.7% inactivation); although these studies used different viral lineages, their results are consistent with the findings in our study (6, 19). The higher inactivation efficiency of the DUV-LED against virus aerosols than against virus suspension might be due to the lower absorption of LED light by the solvent in the virus suspension. Further analyses are required to fully explore the inactivation mechanisms involved.

10.1128/msphere.00941-21.3 FIG S3 Particle image velocimetry of the aerosols in the synthetic quartz tube. See Movie S1 first to assist understanding. The effect of LED light on SARS-CoV-2 in aerosols was evaluated by irradiating the aerosols passing through the synthetic quartz tube with DUV-LED light from outside the tube. Since the time for the aerosol to pass through the irradiation area of the LED light changed depending on the suction flow rate of the air sampler, the irradiation time was regulated by adjusting the flow rate. We visualized the aerosols passing through the tube by using a droplet/aerosol visualization flow measurement system, and the velocity was measured by means of particle image velocimetry. The velocity vectors of the aerosol flowing in the synthetic quartz tube are shown in each panel (corresponding to the color chart shown in the upper left). The number in the upper left corner of each panel indicates the suction flow rate of the air sampler. The aerosols flowed horizontally in the tube at a constant speed, and there was no turbulence in the tube. Download FIG S3, TIF file, 18.2 MB. Copyright © 2022 Ueki et al. 2022 Ueki et al. https://creativecommons.org/licenses/by/4.0/ This content is distributed under the terms of the Creative Commons Attribution 4.0 International license.

10.1128/msphere.00941-21.6 MOVIE S1 Velocities of aerosols in the synthetic quartz tube. The velocity vectors of the aerosols flowing in the synthetic quartz tube are shown when the air sampler was operated at an air flow rate of 90 L/min. The speed of aerosols is indicated by the length and color of the vectors (corresponding to the color chart shown in the upper left). Download Movie S1, AVI file, 19.1 MB. Copyright © 2022 Ueki et al. 2022 Ueki et al. https://creativecommons.org/licenses/by/4.0/ This content is distributed under the terms of the Creative Commons Attribution 4.0 International license.

## DISCUSSION

Our data demonstrate that a 265-nm single-chip 500-mW DUV-LED irradiation system can rapidly inactivate suspended and aerosolized SARS-CoV-2. UV light at 265 nm has a high absorption efficiency for nucleic acids, and it has been reported that UV-C (253.7-nm) irradiation induces genomic damage in SARS-CoV-2 (20); therefore, the inactivation effect of the DUV-LED on SARS-CoV-2 observed in this study may have been caused by damage to the viral genome rather than to the viral proteins. This compact mercury-free irradiation system thus offers substantial advantages over conventional LEDs and mercury vapor lamps. In addition, our findings show that 265-nm DUV-LED irradiation is approximately nine times more effective against aerosolized SARS-CoV-2 than against SARS-CoV-2 suspension. The 265-nm DUV-LED could be used to disinfect the surface of objects and could be incorporated into air purifiers or air conditioners to achieve rapid inactivation of SARS-CoV-2 in the air, thus contributing to the improvement of public health.

In conclusion, the rapid inactivation and remarkably high inactivation efficiency for aerosolized SARS-CoV-2 shown by this high-power 265-nm DUV-LED will open up new possibilities for its practical application in low-cost, high-efficiency, direct air inactivation systems to control the spread of SARS-CoV-2.

## MATERIALS AND METHODS

### Virus.

The SARS-CoV-2 strain (UT-NCGM02/Human/2020/Tokyo) was propagated in VeroE6/TMPRSS2 (JCRB1819) cells with Dulbecco’s modified Eagle’s medium (DMEM) containing antibiotics as described previously (21). VeroE6/TMPRSS2 cells (22) were obtained from the National Institutes of Biomedical Innovation, Health, and Nutrition, Japan. The cells were maintained in DMEM containing 10% fetal calf serum (FCS) and antibiotics at 37°C with 5% CO_2_.

### DUV-LED irradiation device.

A DUV-LED unit consisting of a 1.8- by 1.8-mm^2^ single-chip LED flip-chip was positioned on a stage whose height could be adjusted in the vertical direction at an angle that allowed light irradiation in the vertical downward direction (see Fig. S1 in the supplemental material). The LED light was focused by collimators, and the height of the sample stage was adjusted to irradiate samples at 54 mW/cm^2^ (diameter, 20 mm). The irradiation time of the LED was controlled by using a bipolar power supply.

### Chamber for DUV-LED irradiation of SARS-CoV-2 aerosols.

To investigate the effect of the DUV-LED on viral aerosols, a test chamber for generating viral aerosols was developed. The test chamber (900 mm long by 400 mm wide by 445 mm high) was made of acrylic panels and constructed in a biosafety cabinet at our biosafety level 3 facility (Fig. S2). One side plate of the chamber was connected to a customized compressor nebulizer (NE-C28; Omron) and released a mist of virus suspension. Although the nebulizer initially sprayed rough droplets of virus suspension, 5 min later, more than 99.9% of the particles drifting in the test chamber were less than 2 μm in diameter and were, therefore, considered to be aerosols (Table S2).

The other side was connected to an air sampler (MD8 Airscan; Sartorius) through a synthetic quartz tube (center diameter, 20 mm) with high DUV transmission performance. The exposure area in the quartz tube was adjusted to an irradiation intensity of 54 mW/cm^2^. The viral aerosols wafting through the chamber were passed through the synthetic quartz tube and trapped by the gelatin membrane (catalog no. 12602-080-ALK; diameter, 8.0 cm; pore size, 3.0 μm; Sartorius AG) of the air sampler.

To evaluate the effect of LED light on virus aerosols, the aerosols passing through the synthetic quartz tube were irradiated with DUV-LED light from outside the tube. Since the time for the aerosol to pass through the irradiation area of the LED light changed depending on the suction flow rate of the air sampler, the irradiation time was regulated by adjusting the flow rate. The aerosols passing through the tube were visualized by using a droplet/aerosol visualization flow measurement system (a custom order; Kato Koken), and the velocity was measured by means of particle image velocimetry. The aerosols flowed horizontally through the tube at a constant speed.

### DUV-LED irradiation of SARS-CoV-2 suspension.

Eighty microliters of virus suspension (2.5 × 10^7^ PFU/mL with 1% bovine serum albumin [BSA]) was spread uniformly in a circular pattern on a glass slide with a water-repellent coating (diameter, 15 mm) on the outer line of the circle. The virus suspension was collected immediately after LED irradiation and assessed by use of a plaque assay to determine the viral titer.

### DUV-LED irradiation of SARS-CoV-2 aerosol.

The nebulizer set up on the test chamber was charged with 5 mL of virus suspension (2.5 × 10^7^ PFU/mL with 1% BSA), and the virus mist was sprayed for 8 min. The aerosols were incubated in the chamber for 5 min and were then collected with the air sampler while being irradiated with DUV-LED light from outside the synthetic quartz tube. The membrane in which the viral particles were trapped was immediately dissolved in 10 mL of DMEM containing 5% FCS at 37°C, and then the virus infectivity was assessed by using a plaque assay, as described previously (23, 24).

### Virus titration assay.

Confluent VeroE6/TMPRSS2 cells in 6-well plates were infected with 200 μL of a dilution of the virus suspension. The virus inoculum was removed after incubation for 1 h at 37°C, and then a 1% agarose solution in DMEM was overlaid on the cells. After incubation for 48 h, the agar-covered cells were fixed with 10% neutral buffered formalin. The plaques were counted after removal of the agar.

### Statistical analysis.

Data are presented as the values measured for each experiment (*n* = 3) and the mean. Dunnett’s test was performed, and differences were considered to be statistically significant when the *P* value was less than 0.05.
