# Supplementary figures and images for: A 265-Nanometer High-Power Deep-UV Light-Emitting Diode Rapidly Inactivates SARS-CoV-2 Aerosols
Source: mSphere. 2022 Mar 17;7(2):e00941-21. doi: 10.1128/msphere.00941-21 (PMC9044969; doi:10.1128/msphere.00941-21)

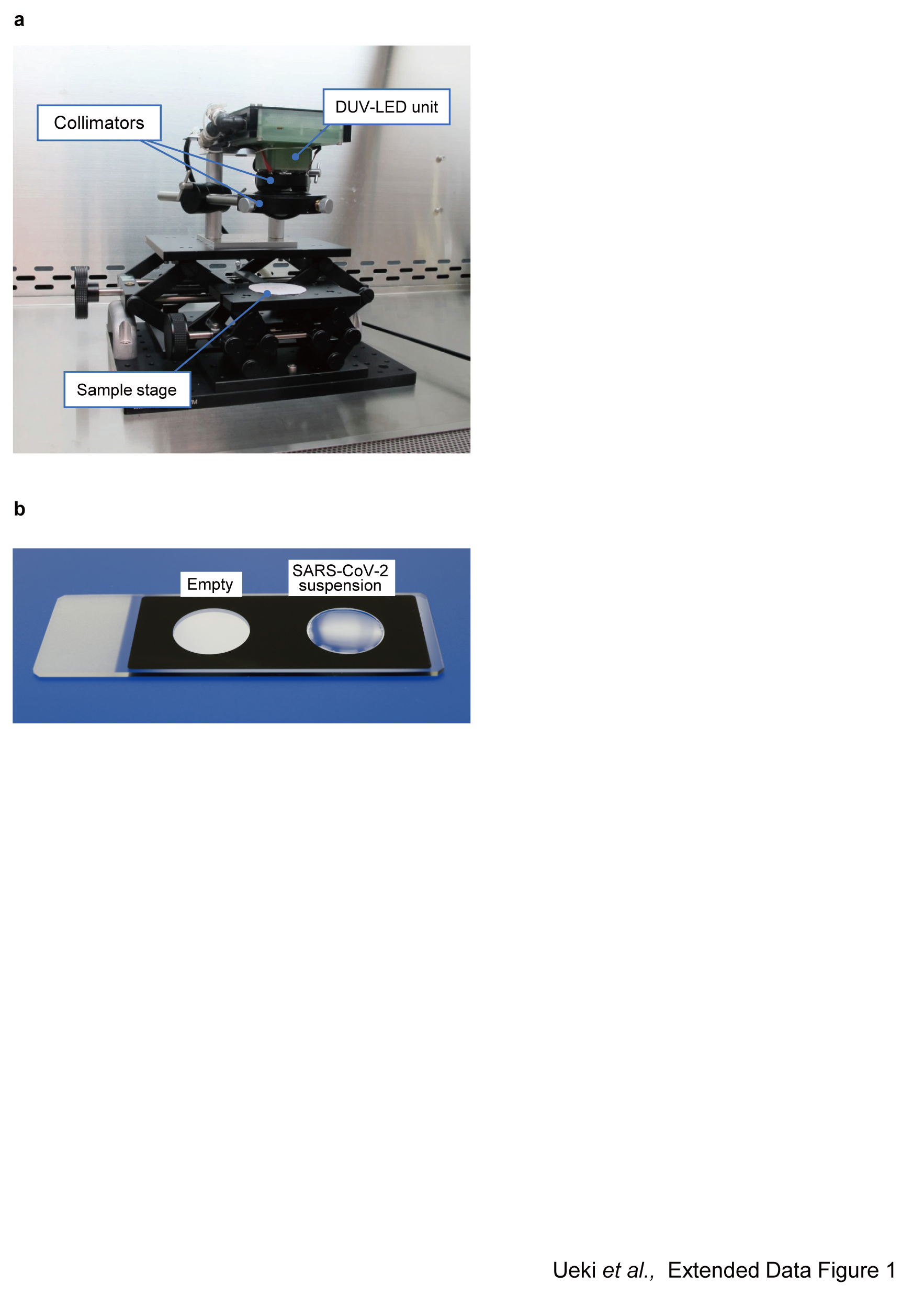

Supplement: FIG S1 [file msphere.00941-21-sf001.tif]

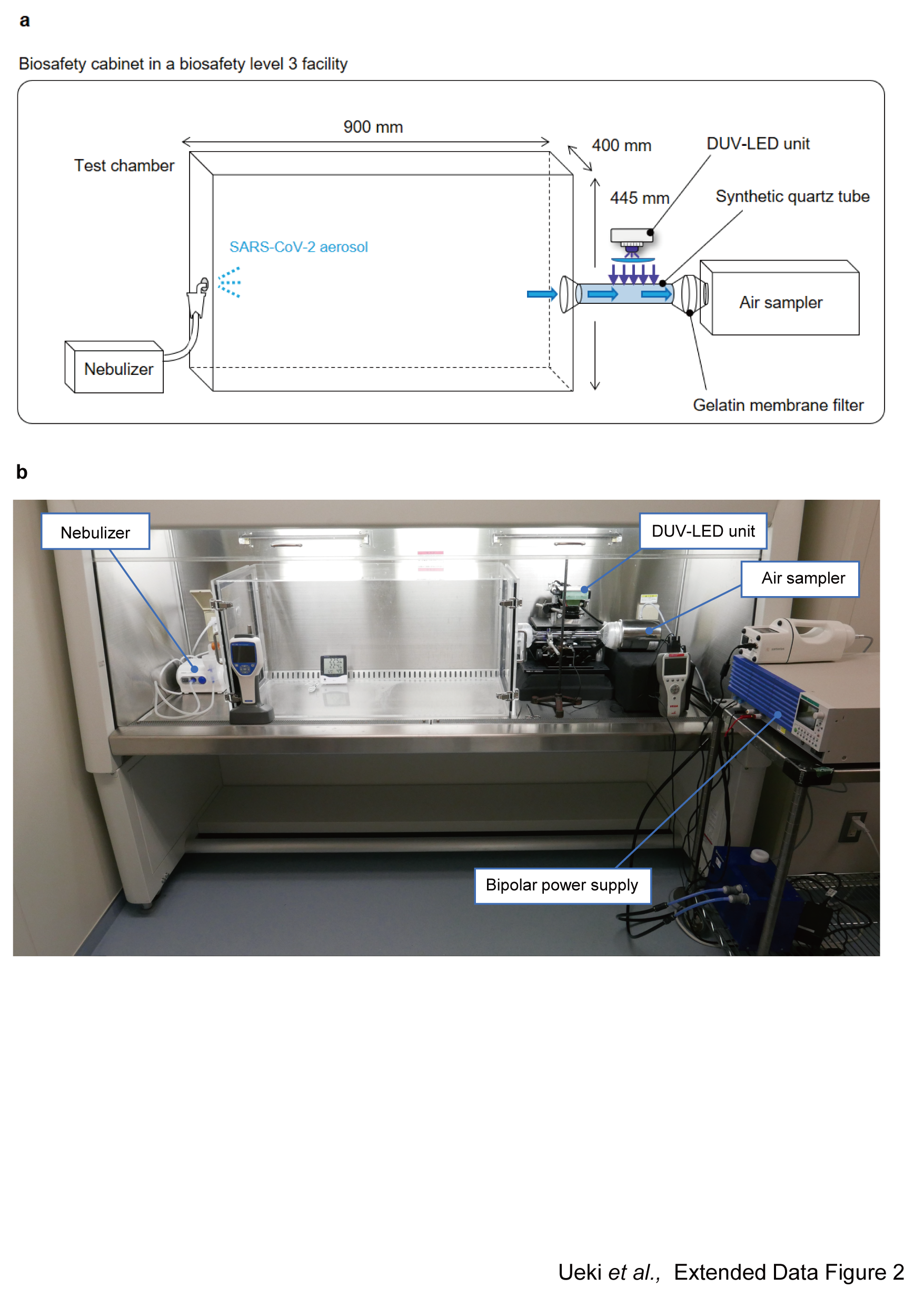

Supplement: FIG S2 [file msphere.00941-21-sf002.tif]

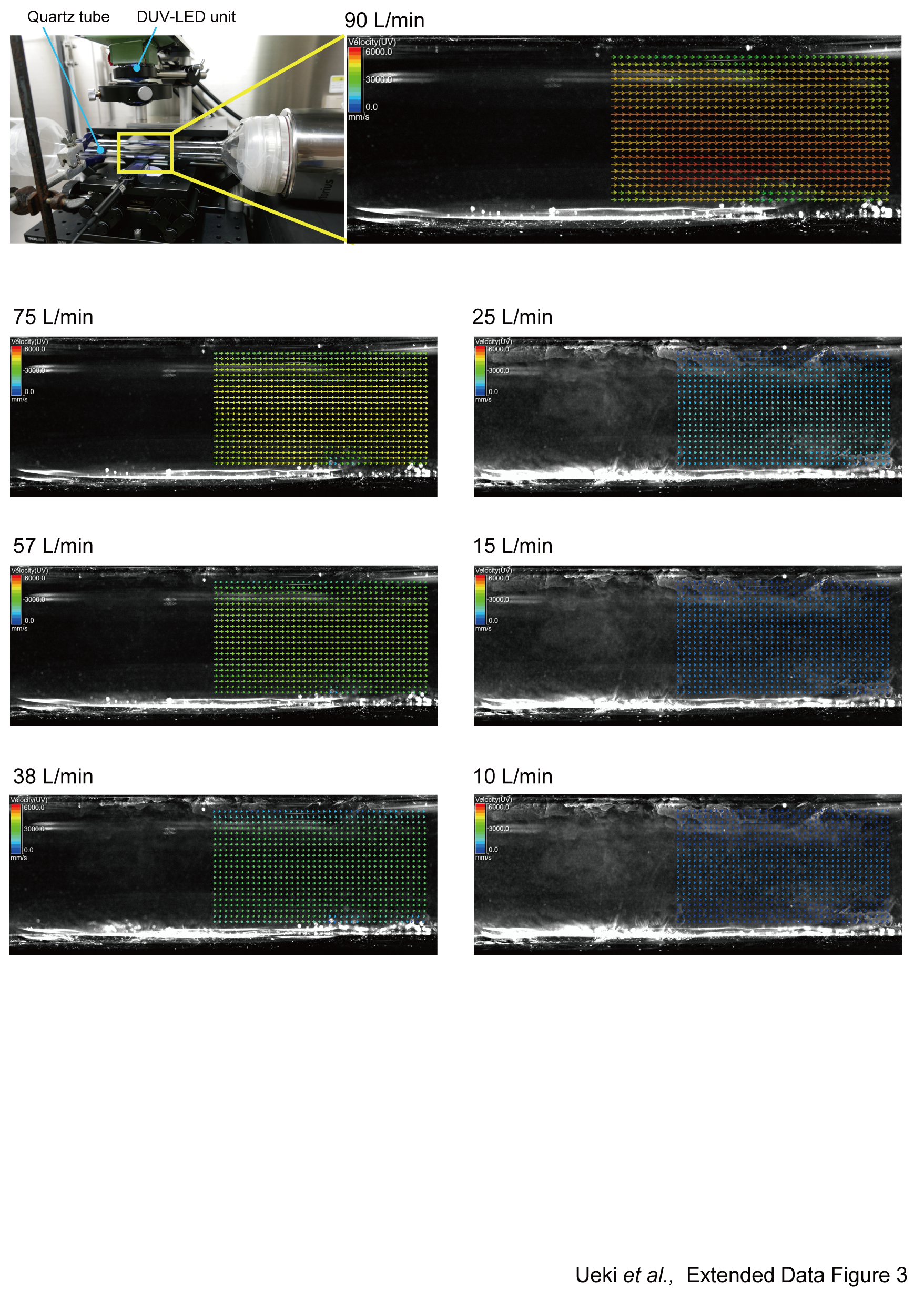

Supplement: FIG S3 [file msphere.00941-21-sf003.tif]
