# Supplementary material for: A 265-Nanometer High-Power Deep-UV Light-Emitting Diode Rapidly Inactivates SARS-CoV-2 Aerosols
Source: mSphere. 2022 Mar 17;7(2):e00941-21. doi: 10.1128/msphere.00941-21 (PMC9044969; doi:10.1128/msphere.00941-21)
Supplement: TABLE S1 [file msphere.00941-21-st001.pdf]

**Supplemental Table S1**

| | | Virus survival rate (%) | | | | |
|---|---|---|---|---|---|---|
| | | 10 | 1 | 0.1 | 0.01 | 0.001 |
| 265 nm DUV-LED irradiation time (s) | Suspension | 0.040 | 0.094 | 0.167 | 0.270 | 0.387 |
| | Aerosol | 0.0043 | 0.0074 | 0.0193 | - | - |
| 265 nm DUV-LED total dose ($mJ/cm^2$) | Suspension | 2.16 | 5.08 | 9.02 | 14.58 | 20.90 |
| | Aerosol | 0.23 | 0.40 | 1.04 | - | - |
| The ratio of the irradiation doses required to achieve each endpoint (Suspension/Aerosol) | | 9.39 | 12.7 | 8.67 | - | - |
