# Supplementary material for: A 265-Nanometer High-Power Deep-UV Light-Emitting Diode Rapidly Inactivates SARS-CoV-2 Aerosols
Source: mSphere. 2022 Mar 17;7(2):e00941-21. doi: 10.1128/msphere.00941-21 (PMC9044969; doi:10.1128/msphere.00941-21)
Supplement: TABLE S2 [file msphere.00941-21-st002.pdf]

	Particle size distribution (µm)					
	0.3-0.5	0.5-1.0	1.0-2.0	2.0-5.0	5.0-10.0	>10.0
Number of particles (count / 0.24L)	76668	51980	22284	8052	70	0
Percentage	48.20	32.68	14.01	5.06	0.04	0.00
